# Huperzine A for Alzheimer’s Disease: A Systematic Review and Meta-Analysis of Randomized Clinical Trials

**DOI:** 10.1371/journal.pone.0074916

**Published:** 2013-09-23

**Authors:** Guoyan Yang, Yuyi Wang, Jinzhou Tian, Jian-Ping Liu

**Affiliations:** 1 Centre for Evidence-based Chinese Medicine, Beijing University of Chinese Medicine, Beijing, China; 2 Department of Neurology (III), Dongzhimen Hospital, Beijing University of Chinese Medicine, Beijing, China; Johns Hopkins Bloomberg School of Public Health, United States of America

## Abstract

**Background:**

Huperzine A is a Chinese herb extract used for Alzheimer’s disease. We conducted this review to evaluate the beneficial and harmful effect of Huperzine A for treatment of Alzheimer’s disease.

**Methods:**

We searched for randomized clinical trials (RCTs) of Huperzine A for Alzheimer’s disease in PubMed, Cochrane Library, and four major Chinese electronic databases from their inception to June 2013. We performed meta-analyses using RevMan 5.1 software. (Protocol ID: CRD42012003249)

**Results:**

20 RCTs including 1823 participants were included. The methodological quality of most included trials had a high risk of bias. Compared with placebo, Huperzine A showed a significant beneficial effect on the improvement of cognitive function as measured by Mini-Mental State Examination (MMSE) at 8 weeks, 12 weeks and 16 weeks, and by Hastgawa Dementia Scale (HDS) and Wechsler Memory Scale (WMS) at 8 weeks and 12 weeks. Activities of daily living favored Huperzine A as measured by Activities of Daily Living Scale (ADL) at 6 weeks, 12 weeks and 16 weeks. One trial found Huperzine A improved global clinical assessment as measured by Clinical Dementia Rating Scale (CDR). One trial demonstrated no significant change in cognitive function as measured by Alzheimer’s disease Assessment Scale-Cognitive Subscale (ADAS-Cog) and activity of daily living as measured by Alzheimer’s disease Cooperative Study Activities of Daily Living Inventory (ADCS-ADL) in Huperzine A group. Trials comparing Huperzine A with no treatment, psychotherapy and conventional medicine demonstrated similar findings. No trial evaluated quality of life. No trial reported severe adverse events of Huperzine A.

**Conclusions:**

Huperzine A appears to have beneficial effects on improvement of cognitive function, daily living activity, and global clinical assessment in participants with Alzheimer’s disease. However, the findings should be interpreted with caution due to the poor methodological quality of the included trials.

## Introduction

Alzheimer’s disease (AD), first described by German psychiatrist Alois Alzheimer in 1906, is a progressive neurodegenerative disease characterized by cognitive deterioration together with behavioral disturbances and declining activities of daily living [1]. It is the leading cause of dementia, resulting in nearly 70% of dementia worldwide by 2005 [2]. An estimated 36 million people [3] worldwide are living with dementia today and more than 115 million is predicted for the year 2050 [4].

Current medications can not cure AD but may help lessen or stabilizes symptoms of AD for a limited time. There are five prescription drugs approved by the U.S. Food and Drug Administration (FDA)-donepezil, galantamine, rivastigmine, tacrine and memantine to treat its symptoms [5]. The first four drugs are cholinesterase inhibitors, which can prevent the breakdown of acetylcholine involved in memory, judgment and other thought progress. Memantine appears to work by regulate the activity of a different chemical messenger in the brain. However, these drugs have some common side effects including nausea, vomiting, loss of appetite and increased frequency of bowel movements, and tacrine is rarely prescribed today because of possible liver damage [5]. Therefore, promising new treatments to slow or stop the progress of AD are urgently needed.

Huperzine A, derived from the Chinese herb *Huperzia serrata*, was identified by scientists in China in the 1980s as a potent, reversible, selective inhibitor of acetylcholinesterase (AChE) [6], which has a mechanism of action similar to donepezil, rivastigmine and galantamine. A large number of preclinical studies and clinical trials had shown the potential effect of Huperzine A in treating AD. A Cochrane systematic review published in 2008 involving 6 randomized trials with 454 AD participants suggested that Huperzine A seemed to have some beneficial effects on AD, however, due to poor methodological quality and small sample size there was still insufficient evidence for clinical recommendation [7]. Another systematic review evaluating single Chinese herbs for AD involving only English-language trials shared similar inconclusive findings [8].

More recently, a variety of clinical trials assessing the efficacy and safety of Huperzine A in treating AD became available. Therefore, we conducted a systematic review to evaluate the beneficial and harmful effect of Huperzine A for treatment of Alzheimer’s disease.

## Materials and Methods

The supporting PRISMA checklist is available as supporting information; see Text S1.

### Standard protocol registrations

This systematic review was registered in PROSPERO, an international prospective register of systematic reviews. The registration identifier of the protocol is CRD42012003249 [9].

### Search strategy and study selection

Two authors (GYY and YYW) searched the following electronic databases from their inception until June 2013: PubMed, Cochrane Dementia and Cognitive Improvement Group and Cochrane Central Register of Controlled Trials (CENTRAL) in the Cochrane Library (April, 2013), China Network Knowledge Infrastructure (CNKI), Chinese Scientific Journals Database (VIP), Wan Fang Database and Sino-Med Database.

We also searched for ongoing trials from mainstream registries: The metaRegister of Controlled Trials [10], ClinicalTrials.gov trials registry [11], The Australian New Zealand Clinical Trials Registry [12], The World Health Organization International Clinical Trials Registry platform [13], and CentreWatch [14].

We also searched unpublished postgraduate theses in Chinese databases. The reference lists of all relevant papers found electronically were hand-searched.

The English searching terms were used individually or combined including “huperzine A”, “Alzheimer’s disease”, “AD”, “Alzheimer disease”, “randomized controlled trial”, “controlled clinical trial”, “randomly”, “trial”, “randomised” and “randomized”. The Chinese searching terms were used individually or combined including those for the generic name of Huperzine A (“*Shi_shan_jian-jia*”), trade names for Huperzine A (“*shuang_yi_ping*”, “*ha_bo_yin*”, “*nuo su lin*”, “*yi nuo*”, “*fu_bo_xin*”, or “*rui li su*”), Alzheimer’s disease (“*lao_nian_chi_dai*”, or “*a_er_zi_hai_mo*”), and randomized (“*sui_ji*”). No language restriction was applied. The detailed search strategy of each database is available as supporting information; see Search strategy S2.

Two authors (GYY and YYW) identified studies that met the inclusion criteria and extracted data independently. The extracted information included: study population, participant demographics and baseline characteristics; details of the intervention and control conditions; study methodology; outcome measures and main results. Any discrepancies were identified and resolved through discussion with a third author (JPL) where necessary.

### Inclusion and exclusion criteria

1. **Type of study.** We included parallel group, randomized control trials to assess the beneficial effects and harms of Huperzine A for treating Alzheimer’s disease, regardless of blinding or publication types. Cross-over randomized trials were also included, but only the outcomes from the first period of treatment were extracted and analyzed. Quasi-randomized trials were excluded.
2. **Type of Participants.** Participants with Alzheimer’s disease regardless of the disease course and severity and diagnosed with any one of the following criteria:
  - The International Classification of Disease (ICD) version 9 or 10;
  - The Diagnostic and Statistical Manual of Mental Disorder (DSM) III, III-R or IV;
  - The National Institute of Neurological and communicative Disorder and Stroke-Alzheimer’s Disease and Related Disorder Association (NINCDS/ADRDA).
  We excluded participants with dementia of other types.
3. **Type of interventions.** Huperzine A for participants with Alzheimer’s disease, regardless of manufactures, preparation form, dose, and duration.
4. **Type of control.** Placebo, no treatment, conventional intervention. We also allowed a Co-intervention if applied in all arms.
5. **Type of outcome measures.** The primary outcomes were cognitive function, and quality of life (QoL) measured by validated instrument. The secondary outcomes were activities of daily living (ADL), global clinical assessment, and adverse effects of Huperzine A.

### Risk of bias (methodological quality) assessment

Two authors (GYY and YYW) independently assessed the risk of bias using the Cochrane of risk of bias tool [15]. The following items were assessed:

- Random sequence generation (selection bias)
- Allocation concealment (selection bias)
- Blinding (performance bias and detection bias)
- Incomplete outcome data (attrition bias)
- Selective outcome reporting (reporting bias)
- Other bias

The risk of bias was categorized as low/unclear/high risk of bias. Trials which met all criteria were judged as having a low risk of bias, trials which met none of the criteria were judged as having a high risk of bias, and trials with insufficient information to judge were classified as unclear risk of bias. Disagreements between the review authors over the risk of bias in specific studies were resolved by discussion and consensus, with involvement of a third review author where necessary.

### Strategy for data synthesis

We performed meta-analyses using RevMan 5.1 software. We summarized data using risk ratios (RR) with 95% confidence intervals (CI) for binary outcomes or mean difference (MD) with 95% CI for continuous outcomes. If different measurement scales were used, standardized mean difference (SMD) analyses were performed. For cross-over trials, only the outcomes from the first period were included. If required data were not reported, we requested data from corresponding author. We used fixed effects model unless there was evidence of heterogeneity. We assessed heterogeneity using both the Chi-squared test and the I-squared statistic. We considered an I-squared value greater than 25% indicative of substantial heterogeneity. We performed funnel plots to detect publication bias.

## Results

### Description of studies

**Figure 1:**
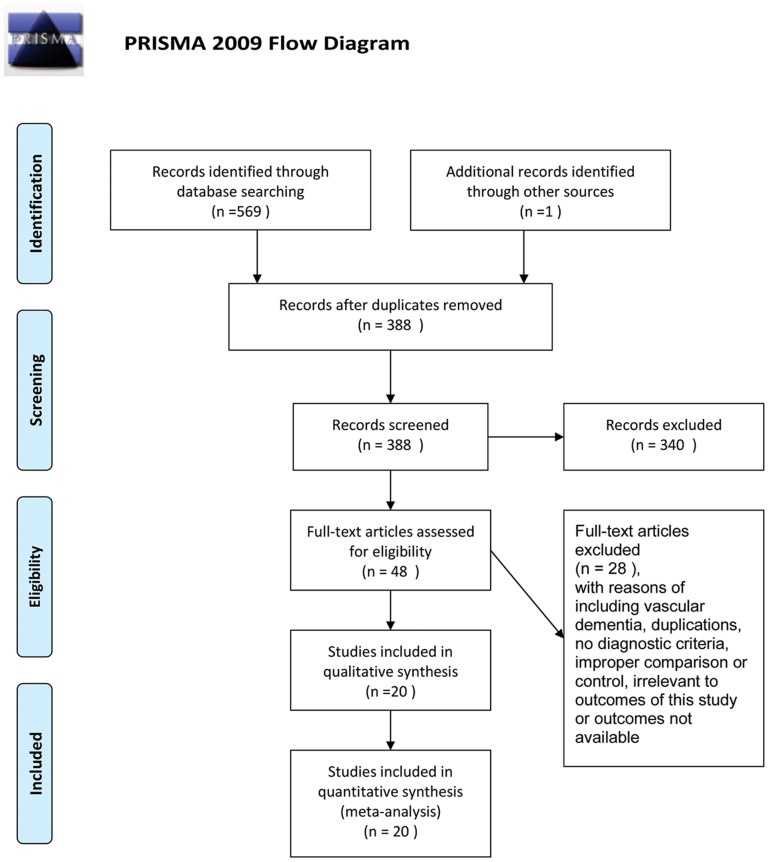
PRISMA flow diagram. Presentation of the procedure of literature searching and selection with numbers of articles at each stage.

A flow chart showed the search process and study selection (Figure 1). We included 20 RCTs [16]–[35] for this systematic review, including five three-armed RCTs [16], [18], [27], [30], [35] and one five-armed RCT [19]. All RCTs were conducted in China, except one which was conducted in the United States [16], and all were published in full: 18 trials [18]–[35] were published in Chinese and two were published in English [16]–[17].

One ongoing registered RCTs was identified from www.clinicaltrials.gov: NCT01282619 evaluating the safety and efficacy of Huperzine A sustained release tablets in patients with mild to moderate Alzheimer’s disease. However, the status was unclear and data were not available.

Table S1 listed the detailed characteristics of the included trials. The 20 RCTs involved 1823 participants with Alzhermer’s disease, aged between 50 to 85 years old. The duration of treatment varied from 8 weeks to 36 weeks, with an average duration of 14.7 weeks. The dosage of Huperzine A varied from 0.2mg to 0.8 mg daily, with an average dose of about 0.37 mg daily.

There are various comparisons: Huperzine A versus placebo (10 trials [16]–[25], 50%), Huperzine A versus no treatment (2 trials [26]–[27], 10%), Huperzine A versus psychotherapy (1 trial [28], 5%), Huperzine A versus conventional medicine (6 trials [29]–[34], 30%), and Huperzine A plus Chinese herbal medicine versus Chinese herbal medicine alone (1 trials [35], 5%).

### Risk of bias assessment

For random sequence generation, four of the 20 trials (20%) [20], [23], [29], [34] used a random number table, one trial (4.17%)[19] used drawing lots, and the other 15 trials (75%) just simply mentioned “randomization” and did not report the specific method of random sequence generation.

For allocation concealment, one trial (5%) [21] used a center controlled method, while the other 19 trials (95%) did not report information on this. One trial [16], though not reporting the method of allocation concealment, assessed the effectiveness of allocation concealment at the end of study by querying clinician, psychometrist, study partner and coordinator. So we judged it as low risk for this item.

For blinding, seven trials (35%) used double-blinding method by blinding of participants and researchers, four trials (16.67%) used single-blinding method in which three reported blinding of outcome assessment and one trial just mentioned “single-blinding”, and the other nine trials (45%) did not give information on blinding. 

For incomplete outcome data, four trials (20%) [16], [19], [21], [23] reported the detailed information of attrition by describing the number and reasons for withdrawal, of which three [16], [19], [23] used intention-to-treat analysis. The remaining trials did not report the information of attrition. Though the number of participants in randomization and in data analysis was equal, it is still unclear whether there is incomplete outcome data in those trials.

For selective outcome reporting, only one trial (5%) [16] was at low risk. This trial gave the clinical trial identifier number, and we were able to find the protocol for comparison and assessment. Since the other 19 trials (95%) did not report the information of registration, we could not make a comparison between the protocols and trial reports.

**Figure 2:**
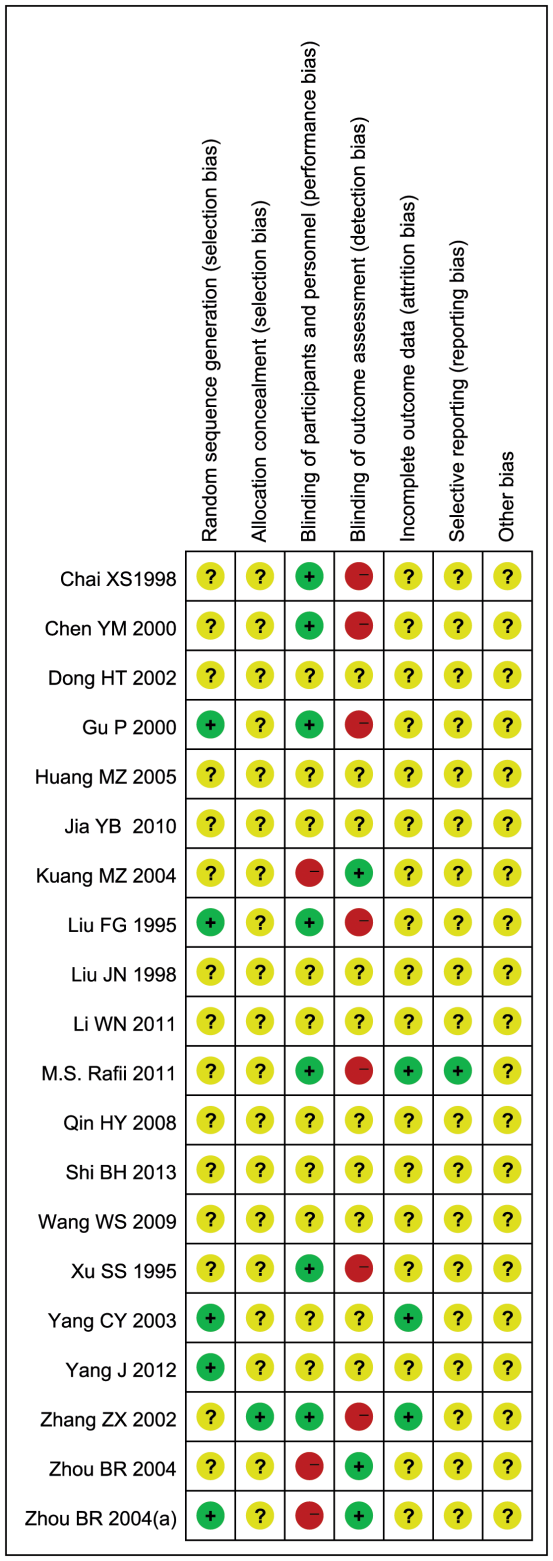
Risk of bias summary. Presentation of the risk of bias summary of the review author’s judgments about each risk of bias item for each included study.

**Figure 3:**
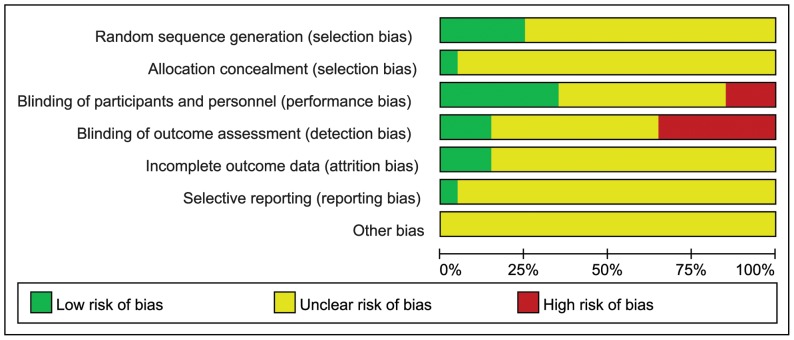
Risk of bias graph. Presentation of the risk of bias graph of the review author’s judgments about each risk of bias item presented as percentages across all included study.

In conclusion, after assessing the selection bias, performance bias, detection bias, attrition bias and reporting bias of the 20 included trials, we found the general methodological quality of most trials was moderate or poor. (Figure 2, 3)

### Effects of interventions

The effect estimates of Huperzine A were shown in the Table S2. We failed to use intention-to-treat analysis, because most included trials did not report the participant flow, especially the number of losses or exclusions after randomization together with reasons.

#### 1. Cognitive function

Compared with placebo, four meta-analyses and most individual trials reporting the last observation scores of cognitive function favored Huperzine A: seven trials favored Huperzine A as measured by Mini-Mental State Examination (MMSE) at 8 weeks [17], [20], [24], 12 weeks [19], [22], [25], and 16 weeks [23], but not at 24 weeks [19]; four trials favored Huperzine A as measured by Hastgawa’s Dementia Scale (HDS) at 8 weeks [17], [20], [24] and 12 weeks [25]; four trials favored Huperzine A as measured by Wechsler Memory Scale (WMS) at 8 weeks [17], [20], [24] and 12 weeks [25]. In trials reporting the change in scores from baseline, when measured by MMSE, three trials showed similar findings favoring Huperzine A at 12 weeks [18], [21], 16 weeks [16], 24 weeks [18] and 36 weeks [18]; however, when measured by Alzheimer’s Disease Assessment Scale-Cognitive Subscale (ADAS-Cog), two individual trials demonstrated there were no statistical differences between Huperzine A and placebo [16], [21].

Compared with no treatment, one trial [26] reporting last observation scores favored Huperzine A as measured by MMSE at 12 weeks, and another trial [27] reporting the change in scores from baseline also favored Huperzine A as measured by WMS.

Compared with psychotherapy, one trial [28] reporting the change in scores from baseline favored Huperzine A when measured by MMSE at 12 weeks.

Compared with conventional medicine, one meta-analyses and all individual trials reporting the last observation scores of cognitive function after treatment favored Huperzine A or demonstrated no statistical difference between groups: as measured by MMSE, Huperzine A resulted in higher scores compared with Piracetam at 8 weeks [30]–[32], but there were no statistical differences between Huperzine A and Galanthamine hydrobromide [29], Vitamin C [33] or Donepezil [34]; as measured by HDS, one trial [29] showed no difference between Huperzine A and Galanthamine hydrobromide at 8 weeks; as measured by WMS, Huerzine A was superior compared with Piracetam at 8 weeks [29] and was not statistically different from Galanthamine hydrobromide at 8 weeks [32].

One trial [35] comparing Huperzine A plus Chinese herbal medicine versus Chinese herbal medicine alone showed no statistical difference between two groups as measured by MMSE at 8 weeks and 12 weeks.

The forest plots of comparison of Huperzine A versus placebo for the outcome of cognitive function as measured by MMSE and activities of daily living as measured by ADL in trials reporting the last observation scores are shown in Figure S1 and Figure S2, respectively.

#### 2. Quality of life (QoL)

No trial reported quality of life.

#### 3. Activities of daily living

Compared with placebo, two meta-analyses and most individual trials reporting the last observation scores favored Huperzine A: seven trials favored Huperzine A as measured by Activities of Daily Living Scale (ADL) at 6 weeks [17], [20], 12 weeks [19], [25], and 16 weeks [23], but not 8 weeks [24] and 24 weeks [18]. In trials reporting the change in scores from baseline, when measured by Alzheimer’s Disease Cooperative Study Activities of Daily Living Inventory (ADCS-ADL), one trial [16] showed no significant change in activities of daily living in Huperzine A group at 16 weeks; however, when measured by ADL, three trials favored Huperzine A at 6 weeks [21], 16 weeks [23], 24 weeks [18] and 36 weeks [18], but not at 12 weeks [18], [21].

No trials comparing Huperzine A versus no treatment reported this outcome.

Compared with psychotherapy, one trial [28] reporting the change in scores from baseline favored Huperzine A as measured by ADL at 12 weeks.

Compared with conventional medicine, three trials reported the last observation scores after treatment on this outcome. One trial [30] demonstrated Huperzine A had a better effect than Piracetam at 8 weeks, but two individual trials showed there were no statistical differences between Huperzine A and Galanthamine hydrobromide [29], or Huperzine A and Donepezil [34].

One trial [35] comparing Huperzine A plus Chinese herbal medicine versus Chinese herbal medicine alone showed no statistical difference between the two groups as measured by ADL at 8 weeks and 12 weeks.

#### 4. Global clinical assessment

One trial [23] reporting the last observation scores demonstrated Huperzine A had a better effect than placebo for the global clinical assessment as measured by Clinical Dementia Rating Scale (CDR) at 16 weeks. No other trials in other comparisons reported this outcome.

A Summary of Finding table presented the main findings of Huperzine A versus placebo for Alzheimer’s disease, and provided key information about the quality of evidence, and a summary of important statistical results on cognitive function, quality of life, activities of daily living and global clinical assessment (Table 1).

**Table 1:** Summary of finding table of Huperzine A versus placebo for Alzheimer's disease.

Outcomes (in trials reporting last observation scores)	Illustrative comparative risks* (95% CI)		No of Participants (studies)	Quality of the evidence (GRADE)	Comments
	Assumed risk (Placebo)	Corresponding risk (Huperzine A)			
**Cognitive function measured by MMSE at 8 weeks**	The mean cognitive function measured by MMSE at 8 weeks ranged across control groups from **12.8 to 17.08 scores**	The mean cognitive function measured by MMSE at 8 weeks in the intervention groups was **3.75 higher** (2.06 to 5.43 higher)	179 (3 studies)	⊕⊕⊕⊖ **moderate^†^**	-
**Cognitive function measured by MMSE at 12 weeks**	The mean cognitive function measured by MMSE at 12 weeks ranged across control groups from **13.5 to 20.39 scores**	The mean cognitive function measured by MMSE at 12 weeks in the intervention groups was **2.89 higher** (1.74 to 4.04 higher)	100 (3 studies)	⊕⊕⊕⊖ **moderate^‡^**	-
**Cognitive function measured by HDS at 8 weeks**	The mean cognitive function measured by HDS at 8 weeks ranged across control groups from **15 to 18.1 scores**	The mean cognitive function measured by HDS at 8 weeks in the intervention groups **was 3.18 higher** (0.30 to 6.06 higher)	179 (3 studies)	⊕⊕⊕⊖ **moderate^†^**	-
**Cognitive function measured by WMS at 8 weeks**	The mean cognitive function measured by WMS at 8 weeks ranged across control groups from **37 to 64.13 scores**	The mean cognitive function measured by WMS at 8 weeks in the intervention groups was **16.77 higher** (10.3 to 23.23 higher)	179 (3 studies)	⊕⊕⊕⊖ **moderate^†^**	-
**Quality of life-not measured**	See comment	See comment	-	See comment	No trials reported this outcome.
**Activities of Daily Living measured by ADL at 12 weeks**	The mean activities of daily living measured by ADL at 6 weeks ranged across control groups from **30.4 to 49.8 scores**	The mean activities of daily living measured by ADL at 6 weeks in the intervention groups was **8.82 lower** (11.47 to 6.16 lower)	70 (2 studies)	⊕⊕⊕⊖ **moderate^‡^**	-
**Global clinical assessment measured by CDR at 16 weeks**	The mean global clinical assessment measured by CDR at 16 weeks in the control group was **2 scores**	The mean global clinical assessment measured by CDR at 16 weeks in the intervention groups was **0.9 lower** (0.98 to 0.82 lower)	65 (1 study)	⊕⊕⊖⊖ **low**¶	-

Presentation of the summary of findings on Huperzine A versus placebo for Alzheimer’s disease in trials reporting the original score after treatment, including information about the review, GRADE of the quality of evidence, and summary of the statistical results information.

Notes: *The basis for the **assumed risk** (e.g. the median control group risk across studies) is provided in footnotes. The **corresponding risk** (and its 95% confidence interval) is based on the assumed risk in the comparison group. †: Two of the three trials did not report the method of randomization and allocation concealment. ‡: One trial just mentioned "randomized" but did not report the method of randomization and blinding. §: The two trials just mentioned "randomized" but did not report the method of randomization. ¶: One trial could not allow to judge inconsistency of results, indirectness of evidence, imprecision and publication bias. **CI:** Confidence interval; **MMSE**: Mini-Mental State Examination; **ADAS-Cog:** Alzheimer’s Disease Assessment Scale-Cognitive Subscale; **HDS:** Hastgawa’s Dementia Scale; **WMS:** Wechsler Memory Scale; **ADCS-ADL:** Alzheimer’s disease Cooperative Study Activities of Daily Living Inventory; **ADL:** Activities of Daily Living Scale; **CDR:** Clinical Dementia Rating Scale.

(GRADE Working Group grades of evidence: we rate study design and specific factors that can downgrade one or two levels of the quality of the evidence including limitations in study design or risk of bias, inconsistency of results, indirectness of evidence, imprecision and publication bias, and factors that can upgrade one or two levels of the quality of the evidence including large magnitude of effect, all plausible confounding would reduce the demonstrated effect or increase the effect if no effect was observed, and dose-response gradient. **High quality:** Further research is very unlikely to change our confidence in the estimate of effect. **Moderate quality:** Further research is likely to have an important impact on our confidence in the estimate of effect and may change the estimate. **Low quality:** Further research is very likely to have an important impact on our confidence in the estimate of effect and is likely to change the estimate. **Very low quality:** We are very uncertain about the estimate.)

#### 5. Adverse events

Of the 20 trials, 7 trials (35%) did not report information on adverse events, one trial (5%) reported no adverse events, and the remaining 12 trials (60%) described the adverse events in detail. The adverse events were mild and included nausea (7/20, 35%), anorexia (loss of appetite) (5/20, 25%), dizziness (4/20, 20%), vomiting (4/20, 20%), constipation (3/20, 15%), insomnia (3/20, 15%), excitability (2/20, 10%), thirst (2/20, 10%), sweating (2/20, 10%), bradycardia (2/20, 10%), abdominal pain (2/20, 10%), somnolence (1/20, 5%), hyperactivity (1/20, 5%), nasal obstruction (1/20, 5%), diarrhea (1/20, 5%), and edema (1/20, 5%).

All of the 13 trials reporting adverse events stated that there were no statistically significant differences between groups on the incidence of adverse events. No trial reported severe adverse events possibly related to Huperzine A.

One trial [16] reported Huperzine A was generally well-tolerated at doses of up to 0.4 mg BID for 24 weeks, even in subjects unable to take other cholinesterase inhibitors.

We conducted funnel plots to detect the publication bias (Figure S3). It demonstrated asymmetrical funnel plots, suggesting potential publication bias.

## Discussion

In this review, we found compared with placebo, Huperzine A demonstrated a potential beneficial effect for Alzheimer’s disease on the improvement of cognitive function as measured by MMSE, HDS and WMS, activities of daily living as measured by ADL and the global clinical assessment as measured by CDR, which is in accordance with the Cochrane reviews published in 2008 [7]; when compared with no treatment, psychotherapy and conventional medicine, the findings also favored Huperzine A.

The 2008 Cochrane review included 6 RCTs comparing Huperzine A with placebo to assess Huperzine A for AD, but reported inconclusive results due to the small sample size and limited methodological quality. Though our review included 10 placebo controlled RCTs (including the six trials included in the 2008 Cochrane systematic review) and another 10 RCTs with other comparisons, we still could not make firm conclusions due to the different measurements (scales) of each outcome, various duration of treatment, and diverse reporting methods in included trials, which resulted in few trials or only one trial in each subgroup. In addition, the methodological quality of most trials was classified as moderate or low, except one phase II trial [16] conducted in United States and published in English.

The following problems in reporting contribute to the limited methodological quality of most included trials: (1) methods of random sequence generation and allocation concealment were not reported; (2) blinding was unclear, if not, who was blinded was unclear; (3) withdrawal/dropout during the trial and use of intention-to-treat analysis was unclear and, if reported, the detailed reasons of withdrawal/dropout were not reported; (4) information on registration was unclear. We highly recommend further randomized trials should report according to the CONSORT Statement [36]. We also recommend further researchers assess allocation concealment and blinding at the end of study, which will be helpful to judge the implementation.

The funnel plot analysis showed asymmetry, which suggests the possibility of publication bias of Huperzine A for AD. The power of the test might be low to distinguish chance from real asymmetry, due to only eight trials included in the funnel plot. However, considering almost all the trials claimed a positive effect of Huperzine A, we still suspected there might be some ‘negative’ studies unpublished. Some researchers suggested prospective registration of clinical trials and/or publication of clinical trial protocol as a solution [37]. In addition, our study might have a limitation of missing some trials since we did not search pertinent English databases such as AMED and EMBASE.

A systematic literature review [38] of 1902 articles identified 68 relevant AD scales. Most scales assessed cognition, activities of daily living and global changes, while other scales assessed behavior/Neuropsychology, participant’s quality of life, and communication/social interaction. In the current review, we also found there were several scales used to measure cognition, activities of daily living and global changes of Alzheimer’s disease in the included RCTs, but no trials reported participant’s quality of life and communication/social interaction, which is important for participants. The trial [16] conducted in the United States reported cognitive function measured by ADAS-Cog and activities of daily living measured by ADCS-ADL and found no beneficial treatment effect of Huperzine A, but did see a positive effect on cognitive function when measured by MMSE. Twenty trials used MMSE alone or in combination of HDS and WMS to measure cognitive function, with one trial [21] using ADAS-Cog and MMSE. We recommend using at least ADAS-Cog and MMSE to measure cognitive function in the future, to improve the reliability and validity.

Though none of the included trials reported severe adverse events possibly related to Huperzine A, we cannot draw firm conclusions about the safety of Huperzine A since seven trials did not report information on safety. The duration of treatment in most trials was 8 weeks or 12 weeks, so the potential beneficial or harmful effect of Huperzine A for treatment of AD might only result from symptomatic changes and short treatment duration, which is consistent with the findings of a previous review [39]. The included trials used different doses of Huperzine A, varying from 0.2mg to 0.8 mg daily. One trial [16] reported Huperzine A was generally well-tolerated at doses of up to 0.4 mg BID for 24 weeks, even in subjects unable to take other cholinesterase inhibitors. Since most participants with Alzheimer’s disease require lifelong treatment, the long-term safety of the treatment is still an important concern. We recommend that further studies should pay attention to the monitoring and reporting of adverse events and long-term safety by designing a longer duration of treatment and a long-term follow-up.

## Conclusions

Huperzine A seems to have some beneficial effects on improvement of cognitive function, daily living activity, and global clinical assessment in participants with Alzheimer’s disease. However, the findings should be interpreted with caution due to the poor methodological quality of the included trials. More rigorous trials are warranted to support its clinical use.

## Supporting Information

Figure S1 **Forest plot of comparison of Huperzine A versus placebo for cognitive function measured by MMSE.**
(TIF)

Figure S2 **Funnel plot of comparison of Huperzine A versus placebo for activities of daily living measured by ADL.**
(TIF)

Figure S3 **Funnel plot of comparison of Huperzine A versus placebo for cognitive function measured by MMSE.**
(TIF)

Table S1 Characteristics of 20 included randomized trials on Huperzine A for Alzheimer’s disease. Presentation of the characteristics of 20 included randomized trials on Huperzine A for Alzheimer’s disease, including study ID, No. of participant, intervention, control, outcome measure and follow-up information.
(DOC)

Table S2 Effect estimates of Huperzine A for Alzheimer’s disease. Presentation of the effect estimates of Huperzine A for the treatment of Alzheimer’s disease, including information about different comparisons under different outcomes.
(DOC)

Text S1 **PRISMA checklist. Presentation of the PRISMA checklist of this systematic review and meta-analysis.**
(DOC)

Text S2 **Search strategy.** Presentation of the detailed search strategy of each database.
(DOC)

Text S3 **Protocol of this systematic review and meta-analysis.** Presentation of the protocol of this systematic review and meta-analysis which registered in PROSPERO.
(PDF)
