# Supplementary figures and images for: Huperzine A for Alzheimer’s Disease: A Systematic Review and Meta-Analysis of Randomized Clinical Trials
Source: PLoS One. 2013 Sep 23;8(9):e74916. doi: 10.1371/journal.pone.0074916 (PMC3781107; doi:10.1371/journal.pone.0074916)

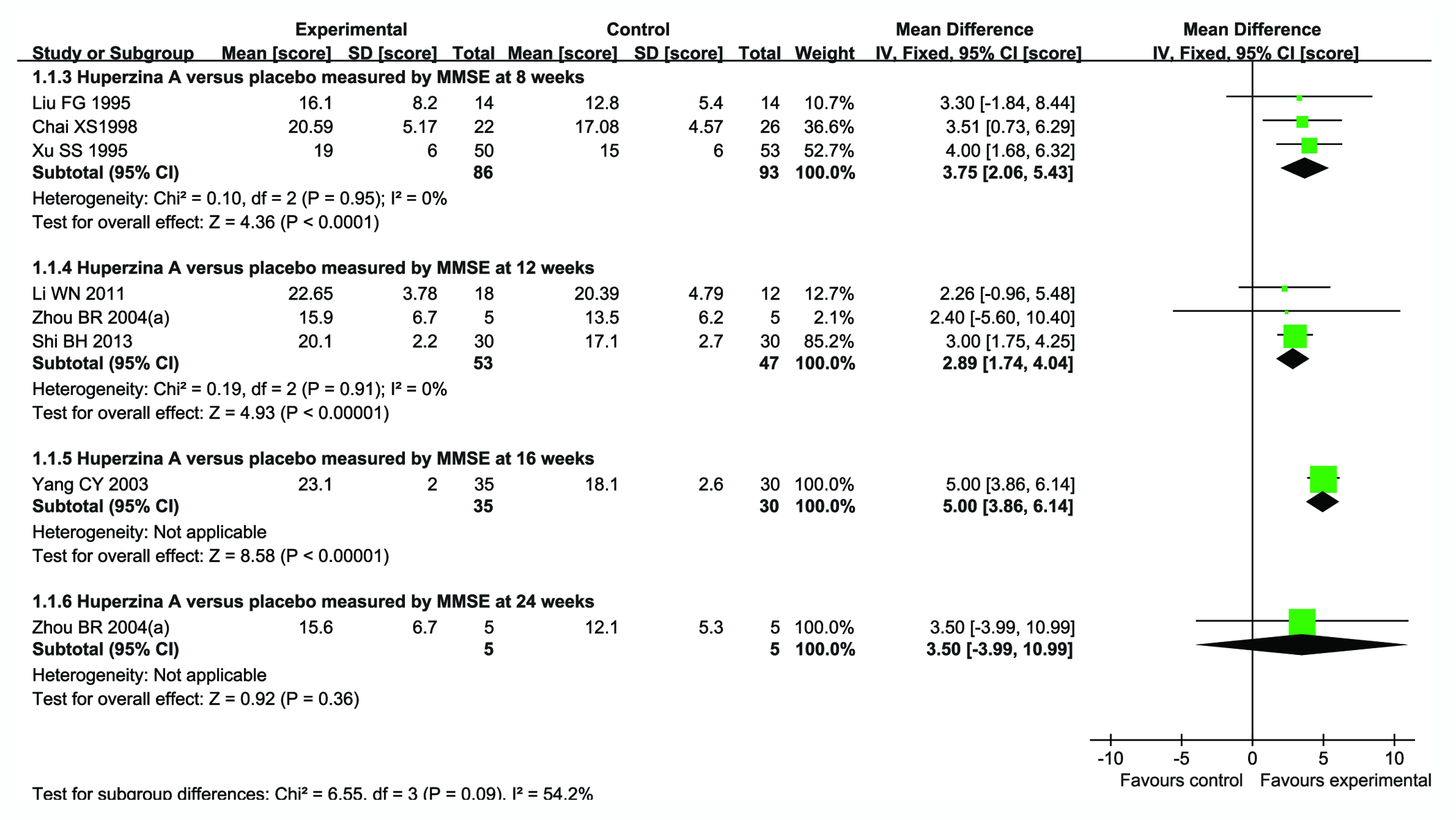

Supplement: Figure S1 — Forest plot of comparison of Huperzine A versus placebo for cognitive function measured by MMSE. (TIF) [file pone.0074916.s001.tif]

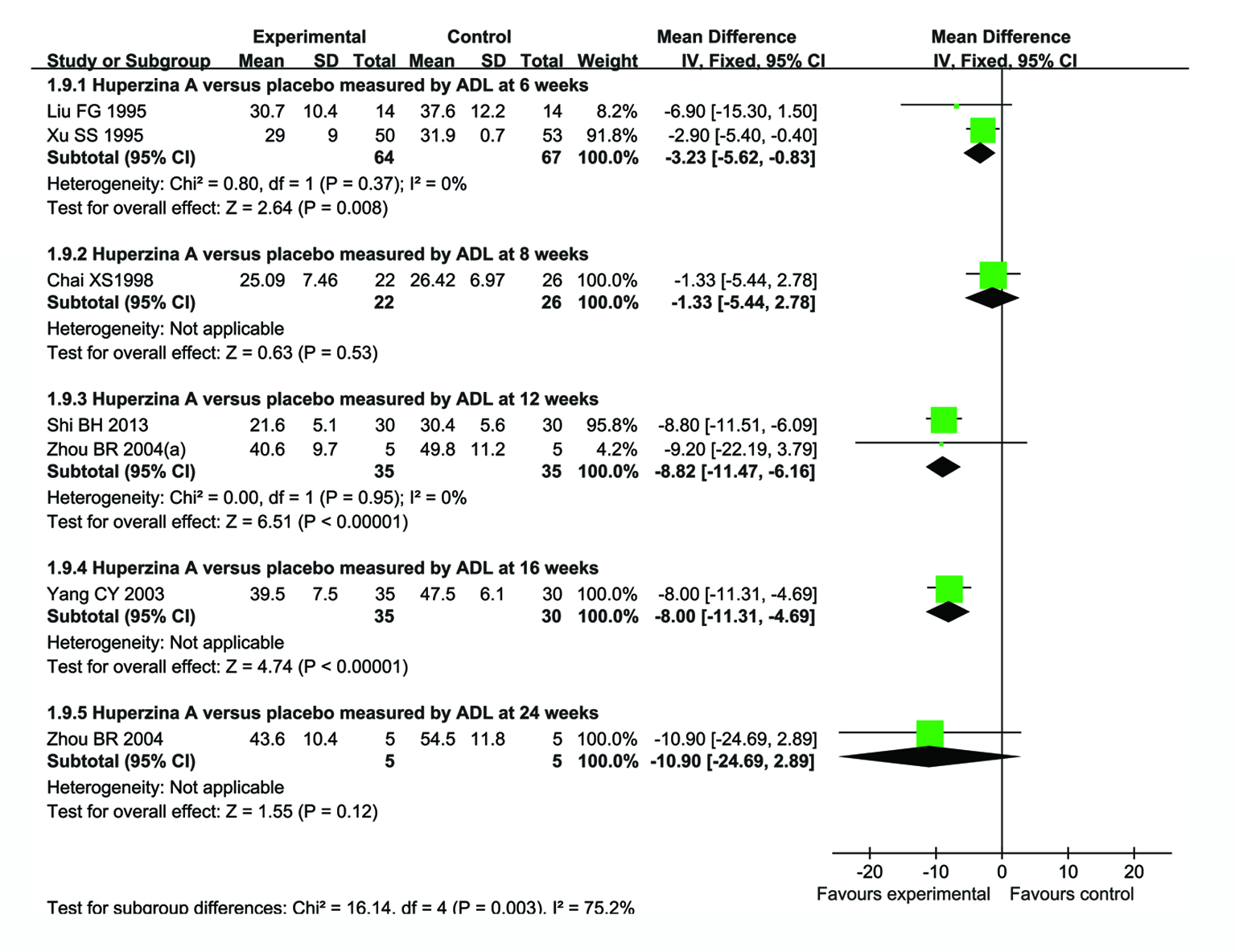

Supplement: Figure S2 — Funnel plot of comparison of Huperzine A versus placebo for activities of daily living measured by ADL. (TIF) [file pone.0074916.s002.tif]

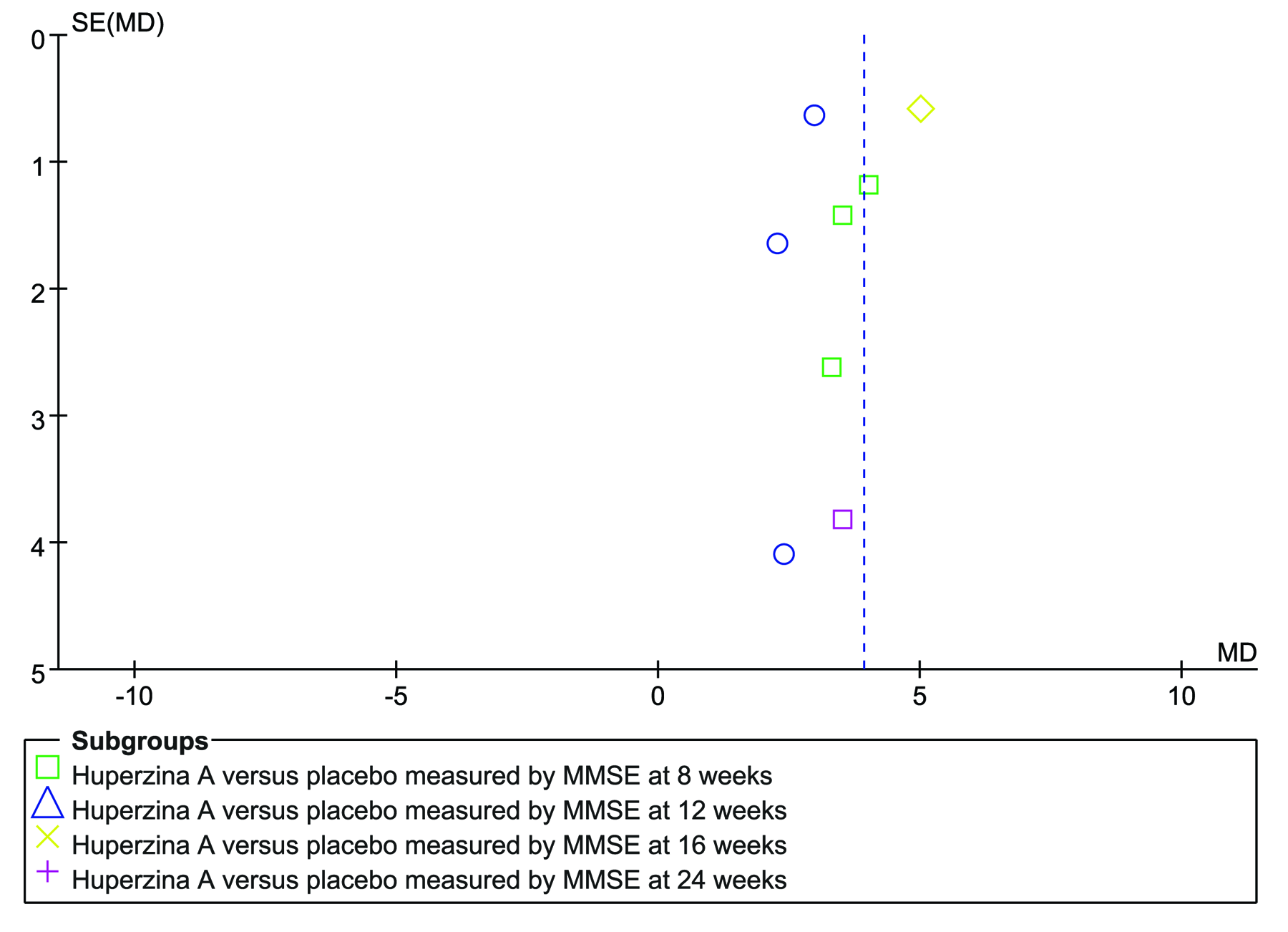

Supplement: Figure S3 — Funnel plot of comparison of Huperzine A versus placebo for cognitive function measured by MMSE. (TIF) [file pone.0074916.s003.tif]
