# Supplementary material for: Huperzine A for Alzheimer’s Disease: A Systematic Review and Meta-Analysis of Randomized Clinical Trials
Source: PLoS One. 2013 Sep 23;8(9):e74916. doi: 10.1371/journal.pone.0074916 (PMC3781107; doi:10.1371/journal.pone.0074916)
Supplement: Table S1 — Characteristics of 20 included randomized trials on Huperzine A for Alzheimer’s disease. Presentation of the characteristics of 20 included randomized trials on Huperzine A for Alzheimer’s disease, including study ID, No. of participant, intervention, control, outcome measure and follow-up information. (DOC) [file pone.0074916.s004.doc]

**Table S1.** Characteristics of 20 included randomized trials on Huperzine A for Alzheimer’s disease.

| **Study ID** | **Sample size** | **Intervention** | **Control** | **Outcome measure** | **Follow-up** |
| --- | --- | --- | --- | --- | --- |
| Rafii 2011^[16]^ | 210 | Huperzine A 0.1mg bid for 2 weeks, then 0.2mg bid for 2 weeks, then 0.3mg bid for 2 weeks, then 0.4 mg bid for 18 weeks, orally | C1: Placebo for 16 weeks then Huperzine A 0.1mg bid for 4 weeks, then 0.2mg bid for 4 weeks;  C2: Huperzine A up to 0. 2mg | ADAS-Cog, MMSE, ADCS-ADL, NPI, CGIC, safety | 8 weeks |
| Xu 1995^[17]^ | 103 | Huperzine A 0.2mg bid for 8 weeks, orally | Placebo 70 mg bid for 8 weeks, orally | WMS, HDS, MMSE, ADL, safety | Not available |
| Zhou 2004^[18]^ | 39 | Huperzine A 0.15mg bid for 36 weeks, orally | C1: Placebo 10mg tid for 36 weeks, orally;  C2:Huperzine A+Aspirin | MMSE, ADL, safety | Not available |
| Zhou 2004a^[19]^ | 25 | Huperzine A 0.15mg bid for 24 weeks, orally | C1:Placebo; C2:Huperzine A+Nicergoline; C3:Huperzine A+Nicergoline+Aspirin;  C4: Huperzine A+ Nicergoline + Aspirin+ estrogen | MMSE, ADL, safety | Not available |
| Liu 1995^[20]^ | 28 | Huperzine A 0.2mg bid for 8 weeks, orally | Placebo 0.2mg bid for 8 weeks, orally | WMS, HDS, MMSE, ADL, safety | Not available |
| Zhang 2002^[21]^ | 202 | Huperzine A 0.1mg bid for one week, then 0.15mg bid for 2 weeks, then 0.2mg bid for 9 weeks, orally; Vitamin E 100mg bid for 12 weeks | Placebo 0.1mg bid for one week, then 0.15mg bid for 2 weeks, then 0.2mg bid for 9 weeks, orally;  Vitamin E bid for 12 weeks | ADAS-Cog，MMSE，ADL，ADSA-non-Cog，CIBIC plus，safety | Not available |
| Li 2011^[22]^ | 30 | Huperzine A 0.15mg bid for 12 weeks, orally | Placebo 3 tablets bid for 12 weeks, orally | MMSE, safety | Not available |
| Yang 2003^[23]^ | 65 | Huperzine A 0.1mg tid for 16 weeks, orally | Placebo 0.1mg tid for16 weeks, orally | MMSE, CDR, ADL, safety | Not available |
| Chai 1998^[24]^ | 48 | Huperzine A 0.2mg bid for 8 weeks, orally | Placebo bid for 8 weeks, orally | WMS, HDS, MMSE, ADL, safety |  |
| Shi 2013^[25]^ | 60 | Huperzine A 0.2mg bid for 12 weeks, orally | Placebo bid for 12 weeks, orally | MMSE, MQ, HDS-R, ADL, safety | Not available |
| Qin 2008^[26]^ | 39 | Huperzine A 0.15mg/d for one week, then 0.25-0.30mg/d for 11 weeks, orally | No treatment | MMSE | Not available |
| Chen 2000^[27]^ | 149 | Huperzine A 0.15-0.2mg bid for 8 weeks, orally | C1:no treatment;  C2: Aniracetam | WMS | Not available |
| Dong 2002^[28]^ | 21 | Huperzine A 0.1mg bid for 12 weeks, orally | Psychotherapy for 12 weeks | MMSE, ADL | Not available |
| Gu 2000^[29]^ | 128 | Huperzine A 0.05mg qid for three days, then 0.1mg qid for 8 weeks, orally; placebo 0.05mg qid for 8 weeks | Galanthamine hydrobromide 5mg qid for 8 weeks;  Placebo 0.05mg qid for 8 weeks | MMSE, WMS, HDS, ADL, safety | Not available |
| Jia 2010^[30]^ | 172 | Huperzine A 0.1mg bid, orally | C1: Piracetam 0.8g tid;  C2:Chinese herbal medicine | MMSE, ADL, safety | Not available |
| Kuang 2004^[31]^ | 61 | Huperzine A 0.1 mg tid for 12 weeks, orally | Piracetam 1.2g tid for 12 weeks, orally | MMSE, safety | Not available |
| Liu 1998^[32]^ | 41 | Huperzine A 0.15mg bid for 8 weeks, orally | Piracetam 1.2g bid for 8 weeks, orally | WMS, MMSE | Not available |
| Huang 2005^[33]^ | 110 | Huperzine A 0.3mg bid for 24 weeks, orally | Vitamine C for 24 weeks, orally | MMSE | Not available |
| Yang 2012^[34]^ | 200 | Huperzine A 0.1mg bid for 24 weeks, orally;  Conventional therapy | Donepezil 5mg once daily for 24 weeks, orally; Conventional therapy | MMSE, ADL | Not available |
| Wang 2009^[35]^ | 90 | Huperzine A 0.1mg bid for 12 weeks, orally; Chinese herbal medicine 150 ml for 12 weeks, orally | C1: Chinese herbal medicine150 ml, orally;  C2: Huperzine A 0.1mg bid for 12 weeks, orally | MMSE, ADL | Not available |

**Abbreviations:** MMSE, Mini-Mental State Examination; ADL, Activities of Daily Living scale; ADAS-Cog, Alzheimer’s Disease Assessment Scale–Cognitive subscale; ADCS-ADL, Alzheimer’s Disease Cooperative Study Activities of Daily Living scale; IADL, Instrumental Activities of Daily Living scale; ADCS-CGIC, Alzheimer’s Disease Cooperative Study–Clinical Global Impression of Change; CDR, Clinical Dementia Rating scale; CIBIC plus, The New York University-Clinician’s Interview-Based Impression of Change-Plus caregiver input; NPI, Neuropsychiatric Inventory; WMS, Wechsler Memory Scale; HDS, Hasegawa’s Dementia Scale; HDS-R, Revised Hasegawa’s Dementia Scale; ADSA-non-Cog, Alzheimer’s Disease Cooperative Study-non-Cognitive subscale; GBS, Gottfries Brane-Steen scale.
