# Supplementary material for: Huperzine A for Alzheimer’s Disease: A Systematic Review and Meta-Analysis of Randomized Clinical Trials
Source: PLoS One. 2013 Sep 23;8(9):e74916. doi: 10.1371/journal.pone.0074916 (PMC3781107; doi:10.1371/journal.pone.0074916)
Supplement: Table S2 — Effect estimates of Huperzine A for Alzheimer’s disease. Presentation of the effect estimates of Huperzine A for the treatment of Alzheimer’s disease, including information about different comparisons under different outcomes. (DOC) [file pone.0074916.s005.doc]

**Table S2.** Effect estimates of Huperzine A for treatment of Alzheimer’s disease in included trials

| **Outcomes and comparisons** | **Effect estimate (95% CI)** | **Studies** | **Participants** | **Study ID** |
| --- | --- | --- | --- | --- |
| **Cognitive function** |  |  |  |  |
| *Huperzine A versus Placebo in trial reporting original score* |  |  |  |  |
| Measured by MMSE at 8 weeks | MD 3.75 [2.06, 5.43] *∆ | 3 | 179 | Liu 1995^[20]^, Chai 1998^[24]^, Xu 1995^[17]^ |
| Measured by MMSE at 12 weeks | MD 2.89 [1.74, 4.04] *∆ | 3 | 100 | Li 2011^[22]^, Zhou 2004a^[19]^, Shi 2013^[25]^ |
| Measured by MMSE at 16 weeks | MD 5.00 [3.86, 6.14] * | 1 | 65 | Yang 2003^[23]^ |
| Measured by MMSE at 24 weeks | MD 3.50 [-3.99, 10.99] | 1 | 10 | Zhou 2004a^[19]^ |
| Measured by HDS at 8 weeks | MD 3.18 [0.30, 6.06] * ∆ | 3 | 179 | Chai 1998^[24]^, Liu 1995^[20]^, Xu 1995^[17]^ |
| Measured by HDS at 12 weeks | MD 2.10 [0.65, 3.55] * | 1 | 60 | Shi 2013^[25]^ |
| Measured by WMS at 8 weeks | MD 16.77 [10.30, 23.23] *∆ | 3 | 179 | Chai 1998^[24]^, Liu 1995^[20]^, Xu 1995^[17]^ |
| Measured by WMS at 12 weeks | MD 9.90 [1.69, 18.11] * | 1 | 60 | Shi 2013^[25]^ |
| *Huperzine A versus Placebo in trial reporting changed score* |  |  |  |  |
| Measured by MMSE at 11 weeks | MD 0.91 [-0.02, 1.84] | 1 | 141 | Rafii 2011^[16]^ |
| Measured by MMSE at 12 weeks | MD 2.38 [1.73, 3.04] *∆ | 2 | 228 | Zhang 2002^[21]^, Zhou 2004^[18]^ |
| Measured by MMSE at 16 weeks | MD 1.50 [0.48, 2.52] * | 1 | 141 | Rafii 2011^[16]^ |
| Measured by MMSE at 24 weeks | MD 2.48 [1.38, 3.58] * | 1 | 26 | Zhou 2004^[18]^ |
| Measured by MMSE at 36 weeks | MD 2.85 [1.44, 4.26] * | 1 | 26 | Zhou 2004^[18]^ |
| Measured by ADAS-Cog at 6 weeks | MD -1.54 [-5.86, 2.78] ∆ | 2 | 343 | Rafii 2011^[16]^, Zhang 2002^[21]^ |
| Measured by ADAS-Cog at 11 weeks | MD -2.56 [-4.11, -1.01] | 1 | 141 | Rafii 2011^[16]^ |
| Measured by ADAS-Cog at 12 weeks | MD -5.36 [-7.04, -3.68] | 1 | 202 | Zhang 2002^[21]^ |
| Measured by ADAS-Cog at 16 weeks | MD -1.58 [-3.31, 0.15] | 1 | 141 | Rafii 2011^[16]^ |
| *Huperzine A versus no treatment in trial reporting original score* |  |  |  |  |
| Measured by MMSE at 12 weeks | MD 6.50[2.60, 10.40] * | 1 | 39 | Qin 2008^[26]^ |
| *Huperzine A versus no treatment in trial reporting changed score* |  |  |  |  |
| Measured by WMS at 8 weeks | MD 5.00 [1.75, 8.25] * | 1 | 94 | Chen 2000^[27]^ |
| *Huperzine A versus Psychotherapy in trial reporting changed score* |  |  |  |  |
| Measured by MMSE at 12 weeks | MD 5.38 [3.72, 7.04] * | 1 | 21 | Dong 2002^[28]^ |
| *Huperzine A versus conventional therapy in trial reporting original score* |  |  |  |  |
| Huperzine A versus Galanthamine hydrobromide by MMSE at 8 weeks | MD 0.32 [-1.12, 1.76] | 1 | 128 | Gu 2000^[29]^ |
| Huperzine A versus Piracetam by MMSE at 8 weeks | MD 4.83 [1.77, 7.90] *∆ | 3 | 162 | Jia 2010^[30]^, Kuang 2004^[31]^, Liu 1998^[32]^ |
| Huperzine A versus Vitamin C by MMSE at 24 weeks | MD 1.18 [-0.85, 3.21] | 1 | 110 | Huang 2005^[33]^ |
| Huperzine A versus Donepezil by MMSE at 24 weeks | MD 1.54 [-0.23, 3.31] | 1 | 200 | Yang 2012^[34]^ |
| Huperzine A versus Galanthamine hydrobromide by HDS at 8 weeks | MD 0.19[-1.63, 2.01] | 1 | 128 | Gu 2000^[29]^ |
| Huperzine A versus Galanthamine hydrobromide by WMS at 8 weeks | MD 1.59[-2.97, 6.15] | 1 | 128 | Gu 2000^[29]^ |
| Huperzine A versus Galanthamine Piracetam by WMS at 8 weeks | MD 3.66[0.87, 6.45] * | 1 | 41 | Liu 1998^[32]^ |
| *Huperzine A+ Chinese herbal medicine versus Chinese herbal medicine in trial reporting original score* |  |  |  |  |
| Measured by MMSE at 8 weeks | MD -0.60[-2.65, 1.45] | 1 | 60 | Wang 2009^[35]^ |
| Measured by MMSE at 12 weeks | MD -0.10[-2.15, 1.95] | 1 | 60 | Wang 2009^[35]^ |
| **Quality of Life** (no trials) |  |  |  |  |
| **Activities of Daily Living** |  |  |  |  |
| *Huperzine A versus Placebo in trial reporting original score* |  |  |  |  |
| Measured by ADL at 6 weeks | MD -3.23 [-5.62, -0.83] *∆ | 2 | 131 | Liu 1995^[20]^, Xu 1995^[17]^ |
| Measured by ADL at 8 weeks | MD -1.33 [-5.44, 2.78] | 1 | 48 | Chai 1998^[24]^ |
| Measured by ADL at 12 weeks | MD -8.82 [-11.47, -6.16] * ∆ | 2 | 70 | Shi 2013^[25]^, Zhou 2004a^[19]^ |
| Measured by ADL at 16 weeks | MD -8.00 [-11.31, -4.69] * | 1 | 65 | Yang 2003^[23]^ |
| Measured by ADL at 24 weeks | MD -10.90 [-24.69, 2.89] | 1 | 10 | Zhou 2004^[18]^ |
| *Huperzine A versus Placebo in trial reporting changed score* |  |  |  |  |
| Measured by ADCS-ADL at 16 weeks | MD 1.79[-0.93, 4.51] | 1 | 141 | Rafii 2011^[16]^ |
| Measured by ADL at 6 weeks | MD -2.36 [-3.67, -1.05] * | 1 | 202 | Zhang 2002^[21]^ |
| Measured by ADL at 12 weeks | MD -4.95 [-11.05, 1.15] ∆ | 2 | 228 | Zhang 2002^[21]^, Zhou 2004^[18]^ |
| Measured by ADL at 16 weeks | MD -8.00 [-11.31, -4.69] * | 1 | 65 | Yang 2003^[23]^ |
| Measured by ADL at 24 weeks | MD -8.25 [-10.64, -5.86] * | 1 | 26 | Zhou 2004^[18]^ |
| Measured by ADL at 36 weeks | MD -8.41 [-11.07, -5.75] * | 1 | 26 | Zhou 2004^[18]^ |
| *Huperzine A versus Psychotherapy in trial reporting changed score* |  |  |  |  |
| Measured by ADL at 12 weeks | MD 6.76 [4.22, 9.30] * | 1 | 21 | Dong 2002^[28]^ |
| *Huperzine A versus conventional therapy in trial reporting original score* |  |  |  |  |
| Huperzine A versus Galanthamine hydrobromide by ADL at 8 weeks | MD 1.11 [-2.59, 4.81] | 1 | 128 | Gu 2000^[29]^ |
| Huperzine A versus Piracetam by ADL at 8 weeks | MD -9.03[-12.31, -5.75] * | 1 | 60 | Jia 2010^[30]^ |
| Huperzine A versus Donepezil by ADL at 24 weeks | MD -0.66[-2.27, 0.95] | 1 | 200 | Yang 2012^[34]^ |
| *Huperzine A+ Chinese herbal medicine versus Chinese herbal medicine in trial reporting original score* |  |  |  |  |
| Measured by ADL at 8 weeks | MD -3.30[-11.37, 4.77] | 1 | 60 | Wang 2009^[35]^ |
| Measured by ADL at 12 weeks | MD -3.80[-11.89, 4.29] | 1 | 60 | Wang 2009^[35]^ |
| **Global clinical assessment** |  |  |  |  |
| *Huperzine A versus Placebo in trial reporting original score* |  |  |  |  |
| Measured by CDR at 16 weeks | MD -0.90[-0.98, -0.82] * | 1 | 65 | Yang 2003^[23]^ |

**Abbreviations:** CI, confidence interval; *, the effect estimate favors experimental group; ∆, result from Meta-analysis. MMSE, Mini-Mental State Examination; ADL, Activities of Daily Living scale; ADAS-Cog, Alzheimer’s Disease Assessment Scale–Cognitive subscale; ADCS-ADL, Alzheimer’s Disease Cooperative Study Activities of Daily Living scale; CDR, Clinical Dementia Rating scale; WMS, Wechsler Memory Scale; HDS, Hasegawa’s Dementia Scale.
