# Supplementary material for: Huperzine A for Alzheimer’s Disease: A Systematic Review and Meta-Analysis of Randomized Clinical Trials
Source: PLoS One. 2013 Sep 23;8(9):e74916. doi: 10.1371/journal.pone.0074916 (PMC3781107; doi:10.1371/journal.pone.0074916)
Supplement: Text S2 — Search strategy. Presentation of the detailed search strategy of each database. (DOC) [file pone.0074916.s007.doc]

**Text S2 Search strategy**

**Search terms:**

1. The English searching terms were used individually or combined including “huperzine A”, “Alzheimer’s disease”, “AD”, “Alzheimer disease”, “randomized controlled trial”, “controlled clinical trial”, “randomly”, “trial”, “randomised” and “randomized”.
2. The Chinese searching terms were used individually or combined including “*Shi_shan_jian-jia*” (Huperzine A), “*shuang_yi_ping*” (trade name of Huperzine A), “*ha_bo_yin*” (trade name of Huperzine A) , “*nuo su lin*” (trade name of Huperzine A), “*yi nuo*” (trade name of Huperzine A), “*fu_bo_xin*” (trade name of Huperzine A), “*rui li su*” (trade name of Huperzine A), “*lao_nian_chi_dai*” (Alzheimer’s disease), “*a_er_zi_hai_mo*” (Alzheimer’s disease), and “*sui_ji*”(randomized).

**Search strategy of each database:**

**PubMed:**

#1 [Title/Abstract] (“huperzine A”)

#2 [Title/Abstract](“Alzheimer's disease” OR “AD” OR “Alzheimer disease”)

#3 #1 and #2

#4 [All fields]("randomized controlled trial" OR "randomised controlled trial" OR "controlled clinical trial" OR "randomly" OR "clinical" OR "trial" OR "random" OR "randomised" OR "randomized")

#5 #3 and #4

**Cochrane Library:**

#1 [Title/Abstract/Keywords] (“huperzine A”)

#2 [Title/Abstract/Keywords](“Alzheimer's disease” OR “AD” OR “Alzheimer disease”)

#3 #1 and #2

#4 [All text]("randomized controlled trial" OR "randomised controlled trial" OR "controlled clinical trial" OR "controlled clinical trial" OR "randomly" OR "clinical" OR "trial" OR "random" OR "randomised" OR "randomized")

#5 #3 and #4

**China Network Knowledge Infrastructure (CNKI):**

#1 [Abstract] (“*Shi_shan_jian-jia*” (Huperzine A) OR “*shuang_yi_ping*” (trade name of Huperzine A) OR “*ha_bo_yin*” (trade name of Huperzine A) OR “*nuo su lin*” (trade name of Huperzine A) OR “*yi nuo*” (trade name of Huperzine A) OR “*fu_bo_xin*” (trade name of Huperzine A) OR “*rui li su*” (trade name of Huperzine A))

#2 [Abstract]( “*lao_nian_chi_dai*” (Alzheimer’s disease) OR “*a_er_zi_hai_mo*” (Alzheimer’s disease))

#3 #1 and #2

#4 [All fields](“*sui_ji*” (randomized or ramdomised))

#5 #3 and #4

**Chinese Scientific Journals Database (VIP):**

#1 [Title/Keywords] (“*Shi_shan_jian-jia*” (Huperzine A) OR “*shuang_yi_ping*” (trade name of Huperzine A) OR “*ha_bo_yin*” (trade name of Huperzine A) OR “*nuo su lin*” (trade name of Huperzine A) OR “*yi nuo*” (trade name of Huperzine A) OR “*fu_bo_xin*” (trade name of Huperzine A) OR “*rui li su*” (trade name of Huperzine A))

#2 [Title/Keywords]( “*lao_nian_chi_dai*” (Alzheimer’s disease) OR “*a_er_zi_hai_mo*” (Alzheimer’s disease))

#3 #1 and #2

#4 [All fields](“*sui_ji*” (randomized or ramdomised))

#5 #3 and #4

**Wan Fang database:**

#1 [MeSH terms] (“*Shi_shan_jian-jia*” (Huperzine A) OR “*shuang_yi_ping*” (trade name of Huperzine A) OR “*ha_bo_yin*” (trade name of Huperzine A) OR “*nuo su lin*” (trade name of Huperzine A) OR “*yi nuo*” (trade name of Huperzine A) OR “*fu_bo_xin*” (trade name of Huperzine A) OR “*rui li su*” (trade name of Huperzine A))

#2 [Title/Keywords] ( “*lao_nian_chi_dai*” (Alzheimer’s disease) OR “*a_er_zi_hai_mo*” (Alzheimer’s disease))

#3 #1 and #2

#4 [All fields](“*sui_ji*” (randomized or ramdomised))

#5 #3 and #4

**Sino-Med Database:**

#1 [Abstract] (“*Shi_shan_jian-jia*” (Huperzine A) OR “*shuang_yi_ping*” (trade name of Huperzine A) OR “*ha_bo_yin*” (trade name of Huperzine A) OR “*nuo su lin*” (trade name of Huperzine A) OR “*yi nuo*” (trade name of Huperzine A) OR “*fu_bo_xin*” (trade name of Huperzine A) OR “*rui li su*” (trade name of Huperzine A))

#2 [Abstract]( “*lao_nian_chi_dai*” (Alzheimer’s disease) OR “*a_er_zi_hai_mo*” (Alzheimer’s disease))

#3 #1 and #2

#4 [All fields](“*sui_ji*” (randomized or ramdomised))

#5 #3 and #4
