# Supplementary material for: Huperzine A for Alzheimer’s Disease: A Systematic Review and Meta-Analysis of Randomized Clinical Trials
Source: PLoS One. 2013 Sep 23;8(9):e74916. doi: 10.1371/journal.pone.0074916 (PMC3781107; doi:10.1371/journal.pone.0074916)
Supplement: Text S3 — Protocol of this systematic review and meta-analysis. Presentation of the protocol of this systematic review and meta-analysis which registered in PROSPERO. (PDF) [file pone.0074916.s008.pdf]

Huperzine A preparation for Alzheimer’s disease: a systematic review and meta-analysis of randomized clinical trials

Guoyan Yang, Yuyi Wang, Yun Xia, Jinzhou Tian, Jianping Liu

Citation

Guoyan Yang, Yuyi Wang, Yun Xia, Jinzhou Tian, Jianping Liu. Huperzine A preparation for Alzheimer’s disease: a systematic review and meta-analysis of randomized clinical trials. PROSPERO 2012:CRD42012003249 Available from http://www.crd.york.ac.uk/PROSPERO/display_record.asp?ID=CRD42012003249

Review question(s)

What are the effectiveness and safety of Huperzine A preparations for the treatment of Alzheimer’s disease?

Searches

We will search the following electronic databases from inception until October 2012: PubMed, MEDLINE, EMBASE, Cochrane Dementia and Cognitive Improvement Group, and Cochrane Central Register of Controlled Trials (CENTRAL).

The following Chinese databases will also be searched: China Network Knowledge Infrastructure (CNKI), Chinese Scientific Journals Database (VIP), Wan Fang Database and Chinese Biomedicine (CBM).

We will also search for ongoing trials from mainstream registries: The metaRegister of Controlled Trials, The US National Institutes of Health Ongoing Trials Register, The Australian New Zealand Clinical Trials Registry, The World Health Organization International Clinical Trials Registry platform, and CentreWatch.

Unpublished postgraduate theses in Chinese databases will also be searched. The reference lists of all relevant papers found electronically will be hand-searched.

Details of the search strategy will be available from the authors on request. No language restriction will be applied.

Types of study to be included

We will include parallel group, randomized controlled trials to assess the beneficial effects and harms of Huperzine A for treating Alzheimer’s disease regardless of blinding or publication types. Cross-over randomized trials will be included, but only outcomes at the first period of treatment will be extracted and analyzed. Quasi-randomized trials will be excluded.

Condition or domain being studied

Alzheimer’s disease, first described by German psychiatrist Alois Alzheimer in 1906, is a progressive neurodegenerative disease characterized by cognitive deterioration together with behavioral disturbances and declining activities of daily living.

Participants/ population

Inclusion: Patients with Alzheimer’s disease regardless of the disease course and severity and diagnosed with any one of the following criteria:

(1) The International Classification of Disease (ICD) version 9 or 10;

(2) The Diagnostic and Statistical Manual of Mental Disorder (DSM) III, III-R or IV;

(3) The National Institute of Neurological and communicative Disorder and Stroke-Alzheimer’s Disease and Related Disorder Association (NINCDS/ADRDA).

Exclusion: Patients with dementia of other type.

Intervention(s), exposure(s)

Huperzine A for patients with Alzheimer’s disease, regardless of manufactures, preparation form, dose, and duration.

Comparator(s)/ control

Placebo, no treatment, conventional intervention.

Co-intervention is allowed if applied in all arms.

Context
No restriction.

Outcome(s)

Primary outcomes
Cognitive function, quality of life (QoL) measured by validated instrument.

Secondary outcomes
Activities of daily living, global clinical assessment, and adverse effects of Huperzine A.

Data extraction, (selection and coding)
Titles and/or abstracts of studies retrieved using the search strategy and those from additional sources will be screened independently by two review authors to identify studies that potentially meet the inclusion criteria outlined above. The full text of these potentially eligible studies will be retrieved and independently assessed for eligibility by two reviewers. Any disagreement will be resolved through discussion with a third reviewer.

A predesigned form will be used to extract data from the included studies for assessment of study quality and data analysis. Extracted information will include: study population and participant demographics and baseline characteristics; details of the intervention and control conditions; study methodology; outcome measures and result. Two review authors will extract data independently. Any discrepancies will be identified and resolved through discussion with a third author where necessary.

Risk of bias (quality) assessment
Two review authors will independently assess the risk of bias using Cochrane tool of Risk of bias. The following items will be assessed:

• Random sequence generation (selection bias)

•Allocation concealment (selection bias)

•Blinding (performance bias and detection bias)

•Incomplete outcome data (attrition bias)

•Selective outcome reporting (reporting bias)

•Other bias

For each included study we will give a description supporting our judgment for each item. Disagreements between the review authors over the risk of bias in particular studies will be resolved by discussion, with involvement of a third review author where necessary.

Strategy for data synthesis
We will summarize data using risk ratios (RR) with 95% confidence intervals (CI) for binary outcomes or mean difference (MD) with 95% CI for continuous outcomes. If different measurement scales are used, standardized mean difference (SMD) analyses will be performed. For cross-over trials, only the first intervention will be included. If required data are not reported, we will request data from corresponding author. We will use fixed effects model unless there is evidence of heterogeneity.

Heterogeneity will be assessed using both the Chi-squared test and the I-squared statistic. We will consider an I-squared value greater than 50% to be indicative of substantial heterogeneity. We will conduct sensitivity analyses based on study quality, if necessary. Funnel plots will be generated to detect publication bias if sufficient trials are identified.

If appropriate, we will use cumulative meta-analyses. Results for the meta-analyses and cumulative meta-analyses will be visualized by forest plots. We will also tabulate the findings by a Summary of Finding Table. Specifically, we will construct trial sequential analysis (TSA) to evaluate our meta-analyses, which can reveal insufficient information size and potentially false results in meta-analysis.

Analysis of subgroups or subsets
If the necessary data are available, subgroup analyses will be done for different outcome measures or different Huperzine A durations separately.

Dissemination plans
We will submit our findings to high impact journal to publicize the findings internationally.

Contact details for further information
Jianping Liu

Center for Evidence-Based Chinese Medicine

Beijing University of Chinese Medicine

Beijing100029

No. 11 Bei San Huan Dong Lu, Chao Yang District
Beijing, P.R.China
jianping_l@hotmail.com

Organisational affiliation of the review
Center for Evidence-Based Chinese Medicine, Beijing University of Chinese Medicine (BUCM)

Review team
Miss Guoyan Yang, Center for Evidence-Based Chinese Medicine, Beijing University of Chinese Medicine, Beijing, China
Mr Yuyi Wang, Center for Evidence-Based Chinese Medicine, Beijing University of Chinese Medicine, Beijing, China
Miss Yun Xia, Office of Scientific Research, Dongfang Hospital Affiliated to Beijing University of Chinese Medicine, Beijing, China
Professor Jinzhou Tian, Department of Gerontology and Geriatrics, Dongzhimen Hospital Affiliated to Beijing University of Chinese Medicine, Beijing, China
Professor Jianping Liu, Center for Evidence-Based Chinese Medicine, Beijing University of Chinese Medicine, Beijing, China

Anticipated or actual start date
01 May 2011

Anticipated completion date
31 December 2012

Funding sources/sponsors
This review is funded by the Program for Innovative Research Team of Beijing University of Chinese Medicine (2011-CXTD-09). Prof Jinzhou Tian and Jianping Liu were supported by the grant number IRT0810 from the Ministry of Education of China.

Conflicts of interest
None known

Language
English

Country
China

Subject index terms status
Subject indexing assigned by CRD

Subject index terms
Alkaloids; Alzheimer Disease; Humans; Neuroprotective Agents; Sesquiterpenes;

Date of registration in PROSPERO
08 November 2012

Date of publication of this revision
08 November 2012

**Stage of review at time of this submission**	**Started**	**Completed**
Preliminary searches	Yes	Yes
Piloting of the study selection process	Yes	Yes
Formal screening of search results against eligibility criteria	Yes	Yes
Data extraction	Yes	No
Risk of bias (quality) assessment	Yes	No
Data analysis	No	No
Prospective meta-analysis	No	No

and registered the review in PROSPERO. CRD bears no responsibility or liability for the content of this registration record, any associated files or external websites.
